# Naked-Eye Detection of Morphine by Au@Ag Nanoparticles-Based Colorimetric Chemosensors

**DOI:** 10.3390/s22052072

**Published:** 2022-03-07

**Authors:** Tahereh Rohani Bastami, Mansour Bayat, Roberto Paolesse

**Affiliations:** 1 Department of Chemical Engineering and Energy, Quchan University of Technology, Quchan 94771-67335, Iran; mansour.chem@gmail.com; 2 Department of Chemical Science and Technologies, University of Rome Tor Vergata, Via Della Ricerca Scientifica 1, 00133 Rome, Italy

**Keywords:** ultrasonic irradiation, morphine, Au@Ag NPs, colorimetric method, naked eye detection, chemosensor

## Abstract

In this study, we report a novel and facile colorimetric assay based on silver citrate-coated Au@Ag nanoparticles (Au@AgNPs) as a chemosensor for the naked-eye detection of morphine (MOR). The developed optical sensing approach relied on the aggregation of Au@Ag NPs upon exposure to morphine, which led to an evident color variation from light-yellow to brown. Au@Ag NPs have been prepared by two different protocols, using high- and low-power ultrasonic irradiation. The sonochemical method was essential for the sensing properties of the resulting nanoparticles. This facile sensing method has several advantages including excellent stability, selectivity, prompt detection, and cost-effectiveness.

## 1. Introduction

Morphine (MOR) is a powerful central nervous system stimulant recommended by the World Health Organization (WHO) in 1998 to relieve cancer-related and severe pains. Today, a large proportion of MOR use has been reported, not as a drug, but as an abused drug. Therefore, the measurement of the MOR level in biological samples can help to determine the level of patients’ treatment, identify the causes of poisoning or death in clinical cases, and control its use/abuse [1].

The exploitation of analytical equipment, such as gas chromatography [2], high-performance liquid chromatography [3], mass spectrometry [4], immunoassay [5], electrochemical [6], and more advanced spectrophotometric methods [7] can allow the qualitative and quantitative detection of MOR with high accuracy and sensitivity.

Combining different methods of chromatography with mass-selective detection is one of the promising trends in the analysis of composite mixtures of unknown compositions, and these techniques are widely used in forensic expert examinations [8].

However, these methods suffer from important drawbacks, because they are bulky, expensive, require high volumes of samples for analysis, trained personnel, as well as long analysis time [9,10].

Chemical sensors can represent a promising solution to these requirements, because they are usually not expensive, simple to use, and can provide sufficient sensitivity and rapid response time, being an effective approach for analytical determination when it is impossible the use laboratory equipment.

Among the different sensor platforms, colorimetric devices based on metallic nanoparticles have been of increasing interest for the direct analysis of materials. These sensors are attractive due to their simplicity, high sensitivity, and low price, making possible in some cases even naked-eye detection.

So far, various colorimetric chemosensors have been developed for MOR detection and, among them, chiral colloidal CdSe quantum dots (CdSe-QDs) functionalized with l- and d-cysteine [11], antimorphine-functionalized graphene quantum dots (GQDs) [12], CdS quantum dots functionalized by antimorphine antibody [13], citrate-capped gold nanoparticles (AuNPs) [14], and melamine modified gold nanoparticles (MA-AuNPs) [15] have been reported.

In particular, silver and gold nanoparticles have been of interest for colorimetric sensor development, due to their chemical stability and their unique surface plasmon resonance (SPR) properties [16,17,18]. The SPR phenomenon facilitates the visual observation of the nanomaterial color changes upon the addition of the target analyte [19,20].

Although Au NPs have been intensively studied, it is important to note that the plasmonic properties of Ag NPs are far superior over Au NPs, due to the near-zero imaginary part of the dielectric function of Ag in a wide wavelength range. Moreover, the real part of the Ag dielectric function is more negative than that of Au [21]. Furthermore, Ag NPs are cheaper and more abundant than Au NPs [21]. Optical excitation of the surface plasmon resonance (SPR) of Ag NPs lies in the visible range at greater molar extinction coefficients and relevant scattering [22].

It is found that colorimetric detection based on the fabrication of core–shell structure in comparison with Au NPs and Ag NPs shows more brilliant color changes. It was suggested that the coating shell onto the inner core led to a change of the local dielectric of environment, the shift of the spectra, and realizing the target molecule detection [23,24]. 

Park et al. applied Au@Ag core–shell nanocubes for colorimetric detection of sulfur ions with a detection limit of 200 ppb and LSPR shifting in the band region of 500 to 800 nm, which showed a rich color change [25]. Gao et al. designed a novel Au@Ag nanorod for the detection of phosphatase activity. This chemosensor could achieve colorimetric detection with high resolution and the color change of the Au nanorod colloids from red to orange–yellow–green–blue–purple is visible to the naked eye [26].

Different methods have been recently developed for the preparation of noble metal nanoparticles, such as physical evaporation–condensation and laser ablation [27,28], chemical reduction [29], microemulsion techniques [30], microwave-assisted synthesis [31], biological method [32], and ultrasonic irradiation [33] can be mentioned. 

Sonochemistry is a prominent approach to reduce metal precursors and produce homogeneous metal nanoparticles with uniform size. The impacts of ultrasound treatment can be assigned to the acoustic cavitation phenomenon. Upon exposure to ultrasound waves, bubbles will form, grow, and collapse in the liquid, leading to the burst of accumulated ultrasonic energy in a very short time. The high local temperature (>5000 K), pressure (>20 MPa), and high cooling rates (>1010 Ks^−1^) are highly appropriate for metal ions. Additionally, the shear forces due to acoustic cavitation can overcome the Van der Waals interactions [34,35,36].

In this work, we propose a mild, inexpensive, and environmentally friendly approach for the preparation of Au@Ag NPs exploited as rapid and sensitive nanoprobes for the naked-eye determination and selective detection of MOR. To the best of our knowledge, no reports on the sonochemical synthesis of chemical nanoprobes have been reported in the literature. In this regard, ultrasonic irradiation (high-power/low-power) was utilized for the synthesis of Au@Ag NPs. In the presence of MOR, the absorbance of Au@Ag NPs increased and a new red-shifted peak appears (620 nm), suggesting the Au@Ag NPs aggregation and increase in NPs concentration in presence of MOR. According to these results, the sonochemical irradiation in the synthetic route was essential for the sensing properties of the resulting nanoparticles. This facile sensing method has several advantages, including excellent stability, selectivity, prompt detection, and cost-effectiveness.

## 2. Materials and Methods

### 2.1. Materials

All reagents were obtained and applied as received without further purification. Trisodium citrate (SC) and glycerol were provided by Merck Company. Silver nitrate (AgNO_3_) and HAuCl_4_ solution (0.1 M, 3.4% *w*/*v*) were freshly bought from Kimia next Company (Isfahan, Iran). Morphine sulfate, acetaminophen (C_8_H_9_NO_2_), methadone (C_21_H_27_NO), methylphenidate (C_14_H_19_NO_2_), and tramadol (C_16_H_25_NO_2_) were obtained from Temad Pharmaceutical Company (Mashhad, Iran). The physicochemical features of morphine are listed in Table S1. Milli-Q water was employed whose minimum resistivity was 18.2 MΩcm^−1^.

### 2.2. Sonochemical Preparation of Silver Citrate Coated Au@Ag NPs

The silver citrate-coated Au@Ag NPs were sonochemically prepared using high- and low-power ultrasonic equipment. Sono-synthesis of Au@Ag NPs was carried under high-power ultrasonic equipment [Au-Ag (P)] using an ultrasonic horn (20 kHz, Branson Digital Sonifier-Connecticut, Danbury, Connecticut, USA, W-450 D) at 50 ± 1 °C for 30 min. Briefly, an aqueous solution including AgNO_3_ (5.0 × 10^−3^ M), HAuCl_4_ (1.0 × 10^−5^ M), trisodium citrate (1.0 × 10^−2^ M), and 3 mL of glycerol was prepared and exposed to ultrasonic irradiation. Different acoustic powers (20 W, 25 W, and 29 W) were tested. After 30 min of sono-reaction, the aqueous solution turned pale-yellow, showing the formation of Au@Ag NPs. The prepared nano-colloid samples were stored at room temperature in darkness; in these conditions, they were stable for longer than four months.

Sono-synthesis of Au@Ag NPs using low power ultrasonic equipment [Au-Ag (B)] was carried out using an ultrasonic bath (40 kHz, 5.7 Li, overall dimensions 325 × 175 × 290 mm; internal dimensions 300 × 153 × 150 mm). Ag/Au solution was similarly prepared, transferred into an Erlenmeyer, and placed in an ultrasonic bath. The temperature was adjusted to 50 ± 1 °C with an aging time of 30 min. The amount of energy transferred to the reaction is low (1–5 Wcm^−2^) [32]. The pale-yellow colloid samples were stored in the same conditions as above, showing similar stability. 

An Au@Ag NPs sample was prepared using the conventional protocol without ultrasound treatment, by stirring an Ag/Au solution for 30 min and 50 ± 1 °C.

### 2.3. General Procedure for Colorimetric Assay of Morphine

Colorimetric detection of MOR was conducted using Au@Ag NPs as nanoprobes. Briefly, 0.5 mL of aqueous solutions with different concentrations of MOR (0–100 µg/mL) were added to 0.5 mL of Au@Ag NPs colloidal solutions and mixed. After 5 min, the change of the solution color was monitored by UV-visible spectroscopy, and photographs were recorded with a digital camera. The solution color from light-yellow turned dark-brown. Transmission electron microscopy (TEM) was used to measure the diameters and morphological changes of Au@Ag NPs in the presence of MOR, which confirmed their aggregation. 

### 2.4. Morphine Assay in Real Sample

MOR-free human urine samples were collected from a healthy volunteer (32-year-old man, 83 kg and 180 cm) according to the institutional guidelines and “Ethics in Medical Research, Handbook of Good Practice”, published by the Center for Research in Medical Ethics and Medical History (Tehran, Iran). The aim of this project was informed to the participant and the consent form was delivered from him. The urine sample was diluted 100-fold with no further pretreatment. For drug determination in human urine, certain levels of MOR were added to all real samples using the standard additive method.

### 2.5. Characterization and Apparatus

The crystal structure of Au@Ag NPs was assessed by Bruker D8-Focus with Cu Kα radiation (λ = 0.154056 nm) in 2θ = 10–80°. Field Emission-Scanning Transmission Electron Microscopy (FE-STEM) images were recorded by Tecnai S-Twin 30, 300 keV, GIF-TRIDIEM. Energy-dispersive X-ray spectroscopy (EDS) results were obtained by the same device. TEM (CM30 Philips, Amsterdam, The Netherlands, 150 kV) was employed to assess the size and morphology of the specimens. Details of the chemical structures were assessed by X-ray photoelectron spectroscopy [XPS; 8025-BesTec XPS system (Berlin, Germany)]. The UV-vis data were recorded by an Evolution 201 Spectrophotometer (Thermo Fisher Scientific, Waltham, MA, USA). IR spectra were obtained by a Thermo Nicolet Avatar-370-FTIR Spectrometer (Thermo Fisher Scientific, Waltham, MA, USA). Zeta-potential assessments were achieved by Zeta Compact (CAD Instrumentation, Île-de, France). Dynamic Light Scattering (DLS) was carried out by Vasco Particle Size Analyzer (Cordouan Technologies, Pessac, France). The high-power ultrasonic equipment operated at 20 kHz and included a double cylindrical jacket (100 mL) for temperature control. The ultrasonic waves were released from the tip at a diameter of 1.1 cm at the end of the horn. The low-power ultrasonic procedure was conducted by Korea RoHs (DSA150-SK) ultrasonic cleaner (40 kHz, 5.7 Li, overall dimensions 325 × 175 × 290 mm, internal dimensions 300 × 153 × 150 mm).

## 3. Results

**Scheme 1:**
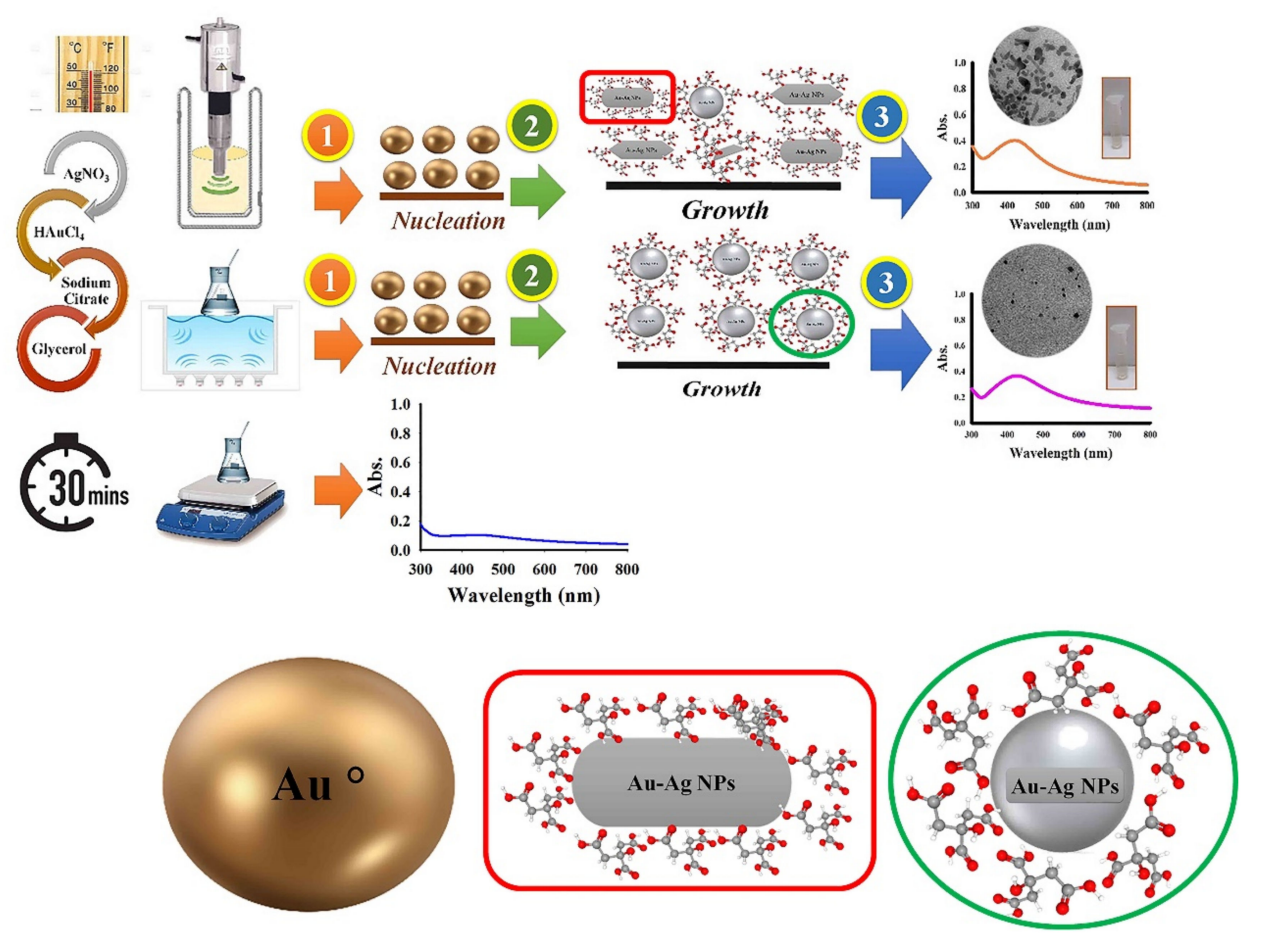
The synthetic strategy for Au@Ag NPs. Step (1) Nucleation of Au° as a seed; Step (2) the nucleation and growth of Ag° onto the surface of the gold seeds; Step (3) UV-vis spectrum of Au@Ag NPs with λ_max_ ~440 nm (In the absence of ultrasonic irradiation (stirrer method), no significant peak was observed).

AgNO_3_ and HAuCl_4_ have been used as precursors for nanoparticle preparation, with citrate as a mild reducing agent and strong complexing stabilizer in Ag and Au reduction [21,37]. The ultrasound-assisted process is depicted in Scheme 1. In the ultrasonic irradiation, with high-temperature (5000 K) local hotspots were formed as a result of the rapid collapse of cavitation bubbles. As a result, the formation of H· [38], and radicals from glycerol pyrolysis occurs [39], and these strong reductants reduce Ag^+^ and Au^3+^ ions. The effect of different parameters such as ultrasonic amplitude (intensity), type of ultrasonic irradiation (direct irradiation/high power/probe type, indirect irradiation/low power/bath type), and types of additives (HAuCl_4_, glycerol) was explored on the synthesis of Au@Ag NPs and their sensor performance.

### 3.1. Optimization of Au@Ag NPs Synthesis Conditions

To optimize the synthesis of Au@Ag NPs, the addition of glycerol, acoustic intensity, and type of ultrasonic irradiation were investigated.

The results obtained are presented in Table 1. The performance of the sensors was evaluated by UV-vis spectra of nanoparticle suspensions, recording the redshifts observed upon addition of MOR (70 µg/mL) at pH = 6.5 (MOR-Au@Ag NPs) after 5 min.

**Figure 1:**
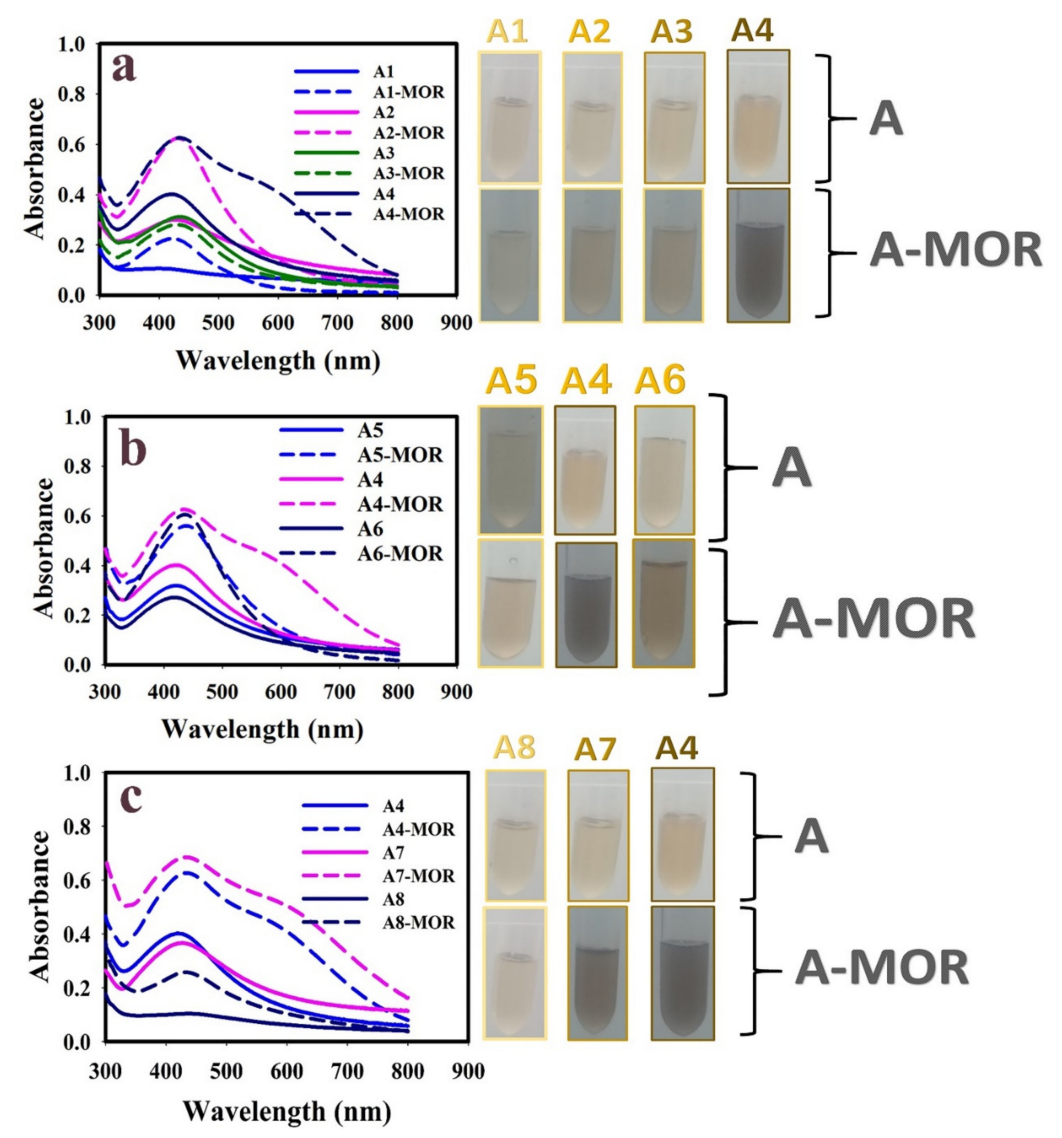
UV-vis spectra of the Au@Ag NPs synthesized under different conditions; (**a**) type of additive, (**b**) acoustic intensity, and (**c**) reaction conditions.

As shown in Figure 1 and Table 1, higher redshifts were observed in A4, A6, A7 samples in presence of MOR. The absorption maxima of metallic NPs are known to shift toward a longer wavelength upon the NPs growth. Therefore, the observed redshift in the Au@Ag NPs SPR peak upon MOR addition indicated the NPs aggregation. It is interesting to note that both HAuCl_4_ and glycerol are necessary to induce a significant redshift and consequently an evident color change of the Au@Ag NPs suspensions. Therefore, the protocol using HAuCl_4_ and glycerol additives, probe-type ultrasound synthesis at the intensity of 26 W/cm^2^, and bath type treatment were selected for subsequent experiments (samples A4 and A7).

The addition of HAuCl_4_ and glycerol led to an increase in NPs size, evidenced by a shift to a longer wavelength [40,41]. The formation mechanism of Au@Ag NPs in presence of Au^3+^ and glycerol can be expressed as follows: first, at pH ~6.0 ± 0.5 the particles are formed through the AuCl_2_(OH)^2−^ and AuCl(OH)^3−^ reduction, with a nucleation time of ~60 s followed by a low-pace growth [42]. The nucleation of Ag° from Ag^+^ in solution occurred onto the surface of the gold seeds, making the particles bigger via Ostwald ripening process. Glycerol can act as a binder to induce coalescence between particles and help the growth process. 

In the absence of ultrasonic irradiation, no significant peak was observed. Therefore, it is suggested that ultrasonic irradiation accelerated the nucleation and growth process and increase the reaction rate leading to the rapid generation of NPs [43]. In addition, in the presence of high-power ultrasonic irradiation, some elongated shapes were obtained. It is suggested that high-power ultrasonic irradiation leads to enhancement of mass transfer and diffusion rate of precursors and the rapid growth of the active axis of NPs. Scheme 1 shows the synthesis strategy for Au@Ag NPs.

### 3.2. Characterization of the Optimized Au@Ag NPs

The Au@Ag NPs were characterized by different spectroscopic methods. Figure 1a,b depict the XRD patterns of Au@Ag (P) and Au@Ag (B). In both cases, the XRD peaks identified the cubic Ag-Au phase (JCPDS-04-0783).

Some diffraction peaks can be assigned to Au@Ag NPs at 2θ values of 38.9°, 44.3°, 64.5°, and 77.5°, which correspond to (111), (200), (220), and (311) silver-gold-metal nanoparticles according to the Fm-3m space group, respectively [44,45]. However, more crystalline structures were found under low-power ultrasonic irradiation.

The peaks at 28.5°, 31.4°, and 46.97° can be attributed to Ag_3_C_6_H_5_O_7_, which correspond to (211), (220), and (222) trisilver citrate, respectively (JCPDS051-0945). The mild peaks can be attributed to organic residuals of the precursors and glycerol [46]. 

Pyrolysis of glycerol in the presence of ultrasonic irradiation led to the production of some organic impurities. CHNS analysis confirmed the presence of C = 14.4% and H = 1.13% in the NPs.

FTIR spectroscopy was carried out to confirm the formation of Au@Ag NPs under high-power (probe type) and low-power (bath type) ultrasonic treatments [Au@Ag (P), Au@Ag (B)]. 

The FT-IR spectra of samples (Figure 1c) exhibited a broad peak at 3435 cm^−1^, due to the O-H stretching vibrations attributable to the water adsorbed on the sample surface. Additionally, the characteristic peaks at (1047 cm^−1^ and 1045 cm^−1^), and (1653 cm^−1^ and 1644 cm^−1^) are related to C–O symmetric stretching, and C=O stretching vibrations, respectively [32]. Based on Figure 1c, in the case of Au@Ag (P), the peaks appeared at shorter wavelengths, confirming the stronger bonding of functional groups onto Au@Ag (P) surfaces [32]. 

**Figure 2:**
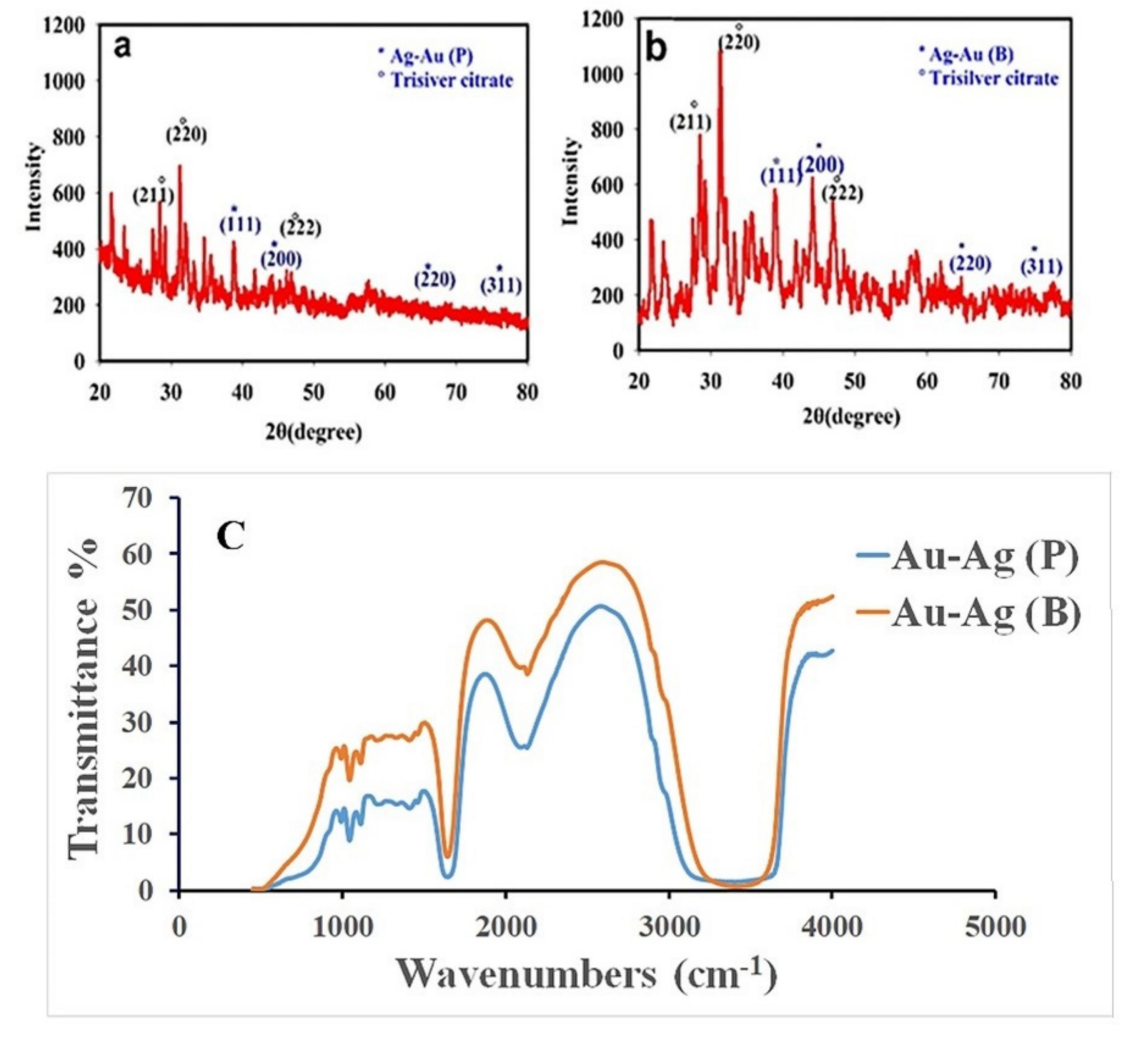
XRD patterns of (**a**) Au@Ag (P); (**b**) Au@Ag (B); and (**c**) FT-IR spectra of Au@Ag (P) and Au@Ag (B).

X-diffraction photoelectron spectroscopy was performed to determine the surface constituent elements of the Au@Ag (P). The sharp peaks in Figure 2a are related to the peaks of C1s, O1s, and Ag3d, due to the presence of carbon, oxygen, and silver with atomic percentages of 72.18%, 18.22%, and 9.60% in the synthesized structure, respectively. In the wide range of C1s, peaks of 284.07, 285.11, 286.29, and 288.65 eV can be assigned to the C-C, C-O, C=O, and O-C=O bands, respectively (Figure 2b). The C-O groups are consistent with the presence of citrate in the sample. In the wide range of O1s, the presence of peaks at 530.41, 532.04, and 532.52 eV can be attributed to metal-oxygen, C=O, and C-O, respectively (Figure 2c). Additionally, in the wide range of Ag3d, characteristic peaks at 369.35 and 375.32 eV confirm the presence of Ag 3d5/2.5 and Ag 3d3/2 on the surface of the composite structure (Figure 2) [47,48].

**Figure 3:**
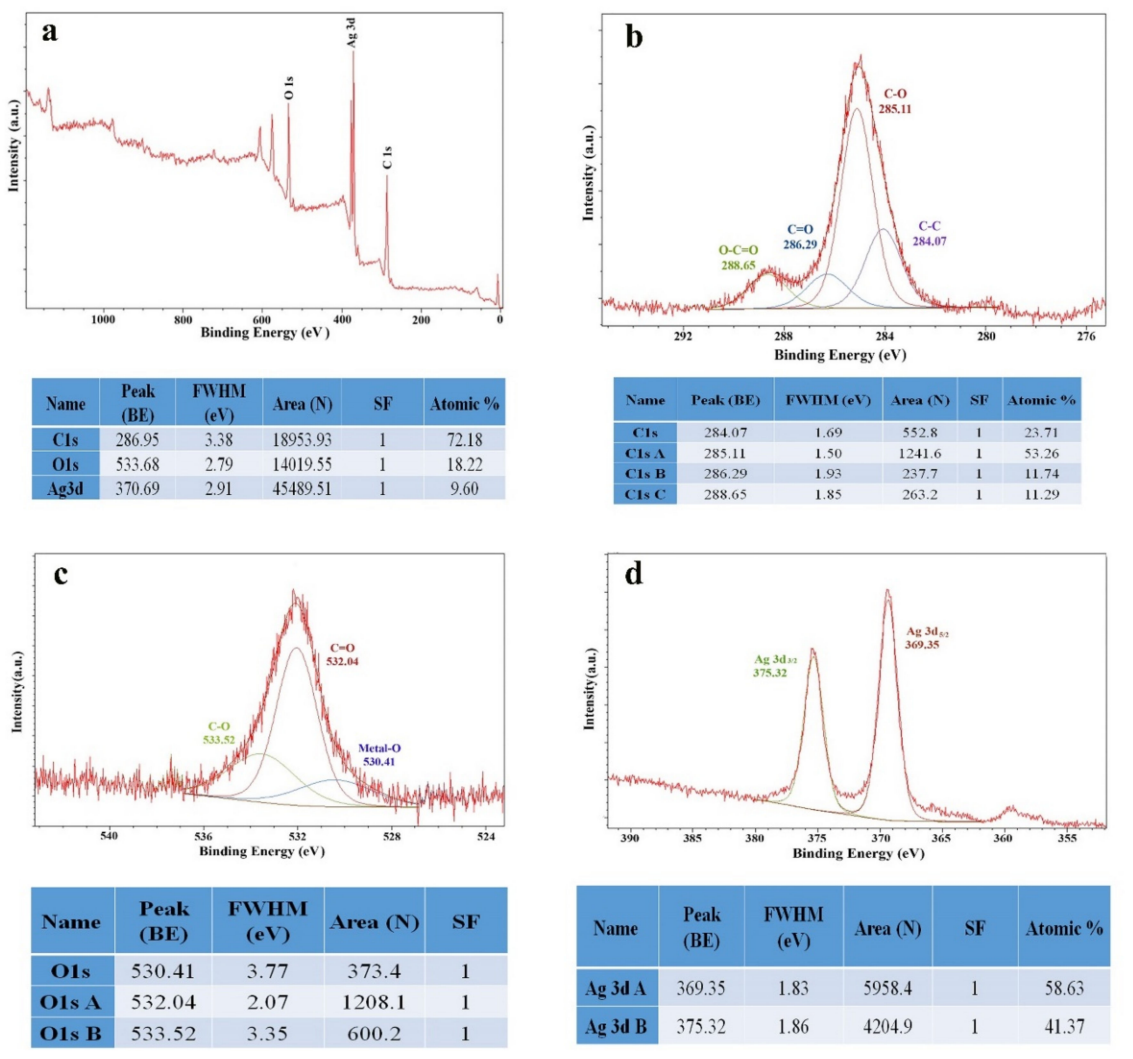
XPS spectra of (**a**) Au@Ag (P); (**b**) C 1s spectrum of Au@Ag (P); (**c**) O 1s spectrum of Au@Ag (P); and (**d**) Ag 3d spectrum of Au@Ag (P).

XPS results indicated no evident peaks related to Au, which further confirms the proposed mechanism of the formation of Au@Ag NPs in presence of Au^3+^. It was suggested that AuNPs act as seeds; due to the high concentration of Ag^+^ in the solution, silver ions are reduced on the surface of gold NPs (Figure 3).

**Figure 4:**
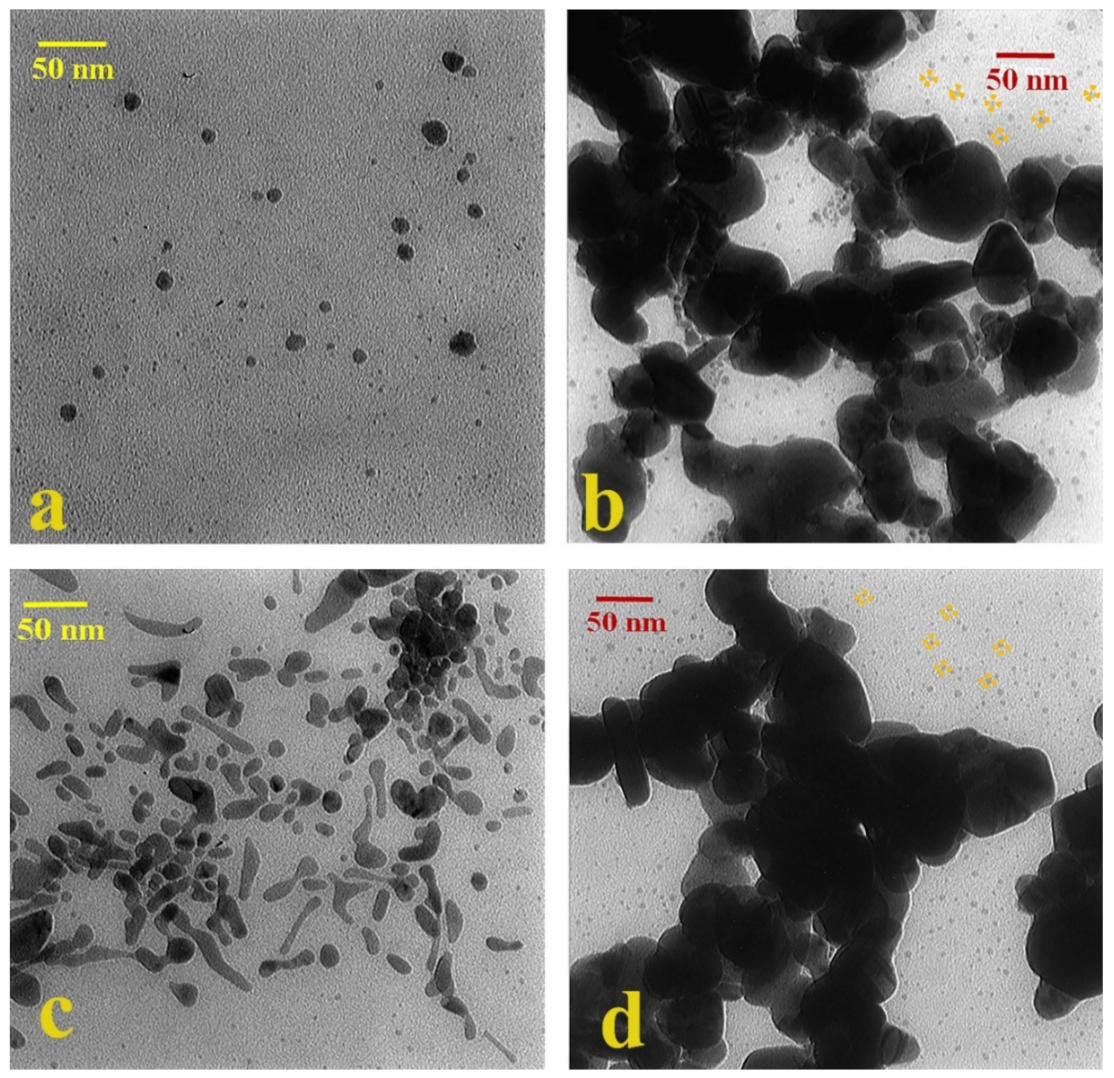
TEM images of (**a**) Au@Ag (B); (**b**) Au@Ag (B)-MOR; (**c**) Au@Ag (P); and (**d**) Au@Ag (P)-MOR.

Figure 4 presents the TEM images of the as-prepared specimens. Based on Figure 4a, spherical Au@Ag NPs were formed with a mean diameter of 5 nm under low-power ultrasonic irradiation (bath type). In the case of Au@Ag NPs synthesized by probe-type ultrasonic irradiation, spherical (mean diameter of 10 nm), and elongated nanocrystals were observed. Most of the elongated particles had a diameter of 8 nm and a length of 28 nm. 

At high-power ultrasonic irradiation, the shock waves and micro-jets induced by the bubble collapse promoted the mass transfer and diffusion of precursors and for this reason, the facets along the more active axis will be quickly grown, leading to the formation of 1D structures [29].

The aggregation of Au@Ag NPs upon exposure to MOR was assessed by TEM because the mean size of aggregated Au@Ag (B) and Au@Ag (P) was 39 and 60 nm, respectively (Table 2). 

The SPR peaks of Au@Ag NPs after the addition of MOR showed a redshift with enhanced absorbance, which confirms the size and concentration of NPs increased. 

After the addition of MOR, some NPs with 4–5 nm in diameter were observed (Figure 3b–d, yellow-marked NPs), confirming the nucleation of new NPs in presence of MOR and a high concentration of Ag^+^.

The aggregation process can be described as follows: hydroxyl groups and furan-like oxygen hybrid rings of MOR can form strong hydrogen bonds with citrate groups present onto the surface of Au@Ag NPs. As a consequence, the aggregation of Au@Ag NPs rapidly occurs, resulting in the color change from yellow to brown and a shift in the plasmon band energy. The other research works confirm the shift in SPR peak into longer wavelength due to aggregation of NPs [49].

The nucleation of NPs can be explained as follows: MOR is an electroactive molecule, that has nitrogen and oxygen atoms, and these atoms are electron-donating sites [50,51]. It is assumed that this electroactivity led to Lewis acid/base interactions between MOR and Ag (I) and Au (III) ions in the colloidal solution of chemical sensors and accelerate the NPs nucleation. The intensity of the SPR peak increased due to an increase in the concentration of NPs.

**Figure 5:**
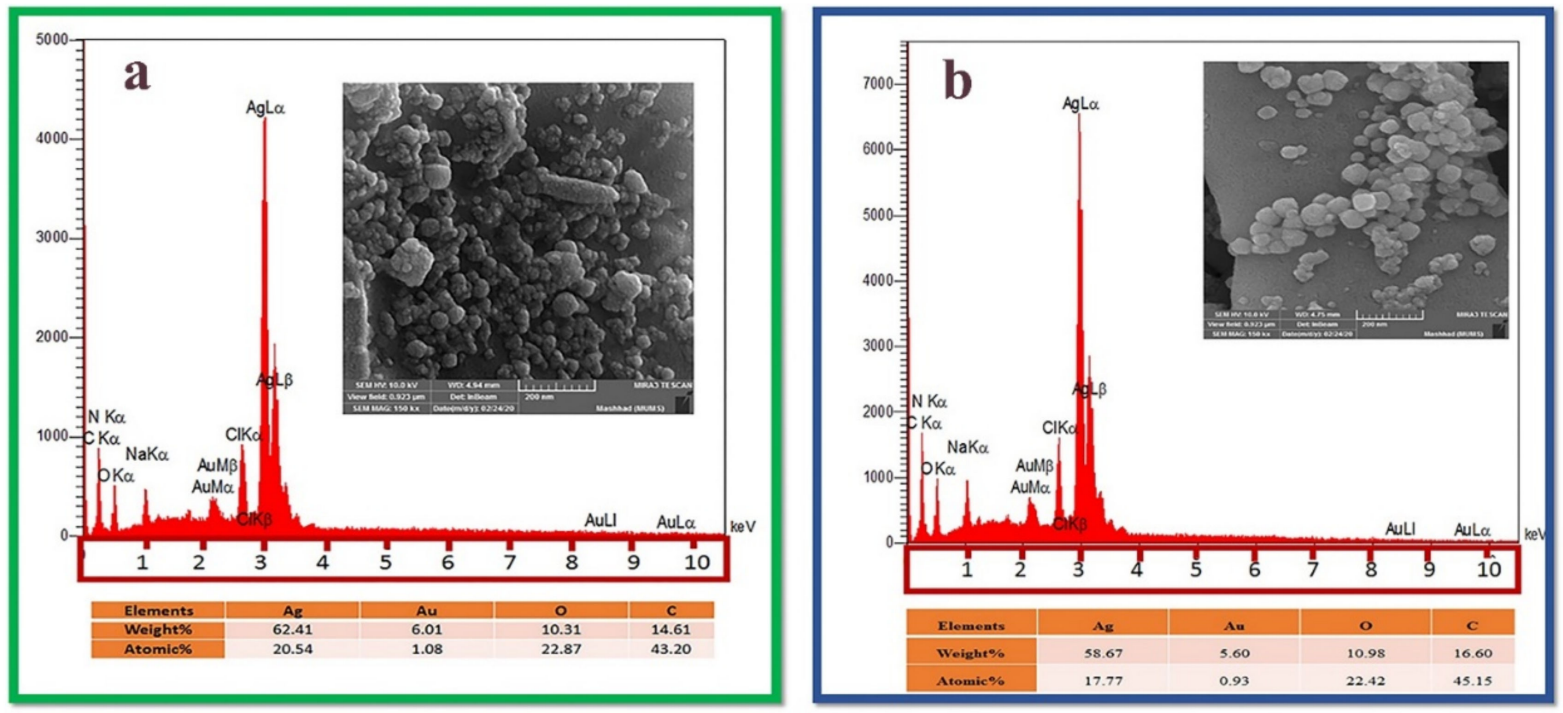
EDX spectrum and chemical composition of (**a**) Au@Ag (P); (**b**) Au@Ag (B).

Figure 5a,b show the EDX spectra of Au@Ag (P) and Au@Ag (B). Ag, Au, C, and O were present in the Au@Ag NPs. C and O peaks are associated with trisilver citrate. These findings verified the formation of Ag NPs with a trace amount of Au. Moreover, the weight percentage of Ag and Au was 62.41% and 6.01% in the Au@Ag (P) and 58.67% and 5.6% in the Au@Ag (B), respectively.

Some impurities like Na, Cl can be observed, with low weight percent that may be due to poor cleaning conditions. 

The ζ potential was measured to assess the surface potential of the as-prepared colloidal Au@Ag NPs suspension at pH = 6.5 ± 0.5 because the ζ potential indicates the suspension stability [52]. The mean ζ-potential of suspensions was −19.3 mV and −23 mV for Au@Ag (P) and Au@Ag (B), respectively. The negative surface charge of NPs can be assigned to the partial ionization of adsorbed citrate. The findings also justified the high colloidal stability (for several months) with no precipitation. The electrostatic repulsions among charged particles promoted particle dispersion with lower aggregation [52]. The FTIR results confirm this assumption. The average ζ-potential of suspensions after the aggregation of Au@Ag NPs in presence of MOR were −15.8 and −16.7 for Au@Ag (P) and Au@Ag (B), respectively. The interaction of MOR on the surface of NPs led to a less negative charge. The pKa for ionization of MOR molecules was 9.9 [53]. Therefore, at pH = 6.5 ± 0.5, MOR is in non-ionized form. Hence, ζ potential of Au@Ag NPs exhibited a lower negative charge after aggregation in the presence of MOR.

The DLS analysis was employed to evaluate the size distribution and hydrodynamic diameter of Au@Ag NPs in the aqueous solution. The average size of Au@Ag (P) and Au@Ag (B) was 39 nm and 36 nm, respectively. The mean size of aggregated Au@Ag (P) and Au@Ag (B) was 114 nm and 380 nm, respectively (Table 2). 

High hydrodynamic diameter confirms the strong interaction of water on the NPs surface. Moreover, the higher dispersion stability can be assigned to the solvation of adsorbed glycerol onto the surface of Au@Ag NPs. As shown in TEM and DLS results, the average sizes of NPs in DLS results are higher than TEM analysis, which is a consequence of the NPs interaction with water molecules. Furthermore, DLS analysis obtains the hydrodynamic radius of NPs while in TEM this hydration layer is not present. As seen in Table 2 the average hydrodynamic size for Au@Ag (B) + MOR is higher than Au@Ag (P) + MOR. It is assumed that due to the stronger shock waves and micro-jets induced by the bubble collapse in high power ultrasonic irradiation (probe type) the adsorbed amounts of glycerol onto the surface of NPs were lower than NPs synthesized using low-power ultrasonic irradiation (bath type). Hence, the interaction of MOR with the surface of Au@Ag (B) was higher and caused a higher hydrodynamic diameter.

### 3.3. Optimization for Colorimetric Assay of Morphine

To optimize the Au@Ag NPs for MOR sensing, two parameters (pH, and incubation time) were investigated.

#### 3.3.1. Effect of Sample pH Value

**Figure 6:**
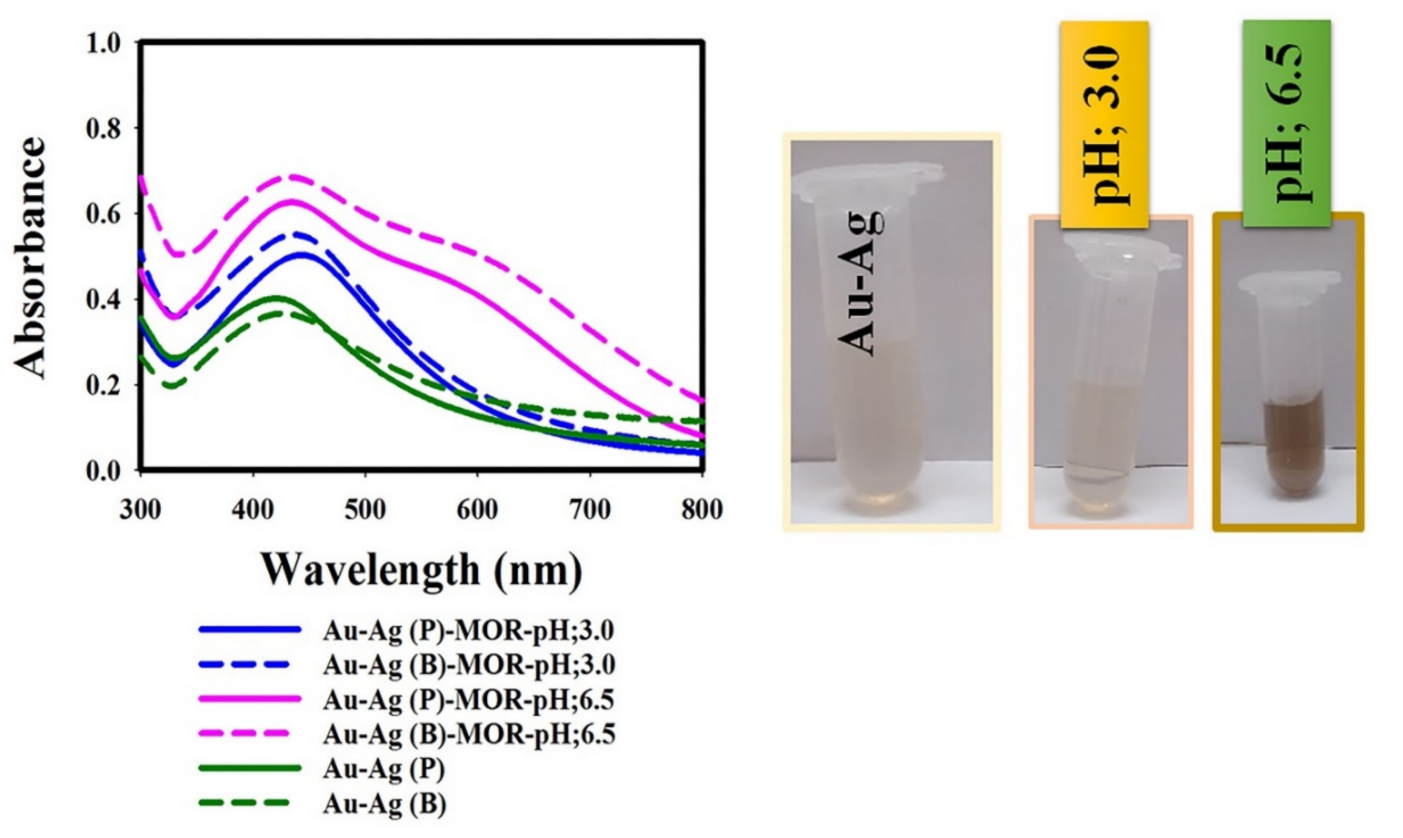
UV-visible absorption spectra of Au@Ag NPs upon addition of MOR solution at different pH. (Photographic images from left to right: Au@Ag NPs + 0 µg/mL (blank), Au@Ag NPs + 70 µg/mL MOR at different pH, incubation time 5 min).

The effect of sample pH on the MOR sensing ability of Au@Ag NPs was explored (Figure 6). The significance of pH lies in the abundance of hydroxyl, carboxyl, and amine groups in drugs [53]. 

The pH of the starting Au@Ag NPs solution was 6.5 ± 0.5; the pH was adjusted to different values by the addition of NaOH and HCl solutions before the colorimetric assay of MOR. The results showed that Au@Ag NPs spontaneously aggregated at pH = 9.0 without the addition of MOR, which caused a dark brown colloidal solution (results not shown). In an alkaline medium, the formation of silver oxide occurs, according to the reaction:

2Ag^+^ + 2 OH^−^ ⇆ Ag_2_O + H_2_O (1)

The formed Ag_2_O deposits onto the NPs surface, removing the negatively charged layer and consequently inducing the NPs aggregation. Thus, a dark-brown color was obtained at higher pH without the addition of MOR. 

As shown in Figure 6, at pH = 6.5 ± 0.5, the intensity of the SPR peaks increased and a peak emerged at longer wavelengths (redshift). Furthermore, a greater color change of Au@Ag NPs from yellow to dark brown was detected. The surface negative charge of Au@Ag NPs at pH = 6.5 ± 0.5 and the positive charge of MOR molecules (pKa of MOR is 9.9) led to the electrostatic attraction and aggregation of nanoparticles. TEM and DLS analysis confirmed the increase in nanoparticle size in the presence of MOR. Additionally, no significant color change and red-shift in the UV-vis spectrum occurred at acidic pH (pH = 3.0). It is known that acidic pH increases the monodispersity of the Ag NPs reducing their concentration. Therefore, pH = 6.5 ± 0.5 was chosen for further studies.

#### 3.3.2. Effect of Incubation Time

**Figure 7:**
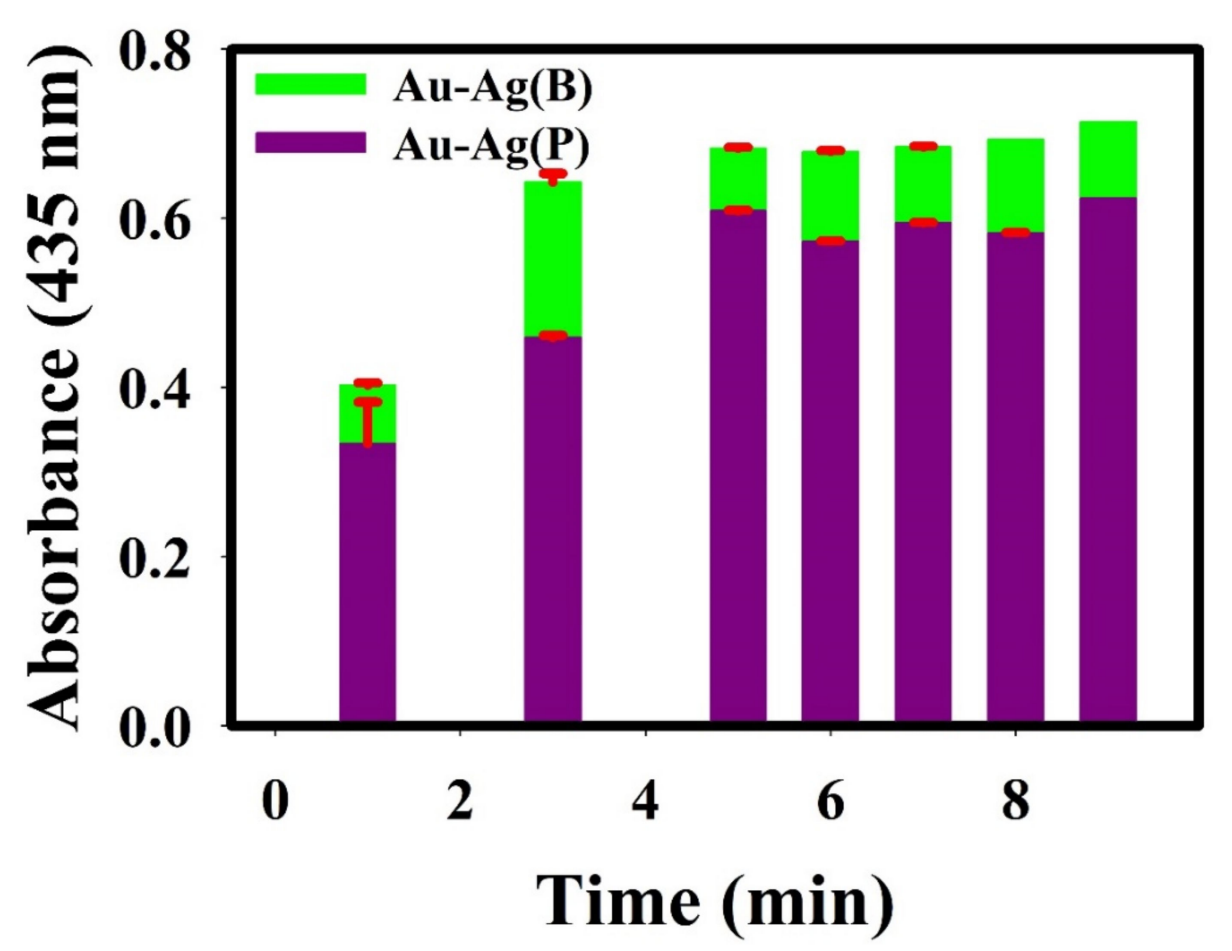
Effect of incubation time for determination of MOR using Au@Ag NPs (condition: pH = 6.5 ± 0.5, MOR conc.; 70 µg/mL).

Various incubation times were evaluated for optimization. The Au@Ag NPs began aggregation immediately after mixing with MOR under optimal conditions, with stable spectral changes within 5 min (the optimal incubation time) (Figure 7). 

According to the results for optimization, pH = 6.5 ± 0.5 and incubation = 5 min were chosen for further studies.

### 3.4. Linear Range of Calibration Curves

**Figure 8:**
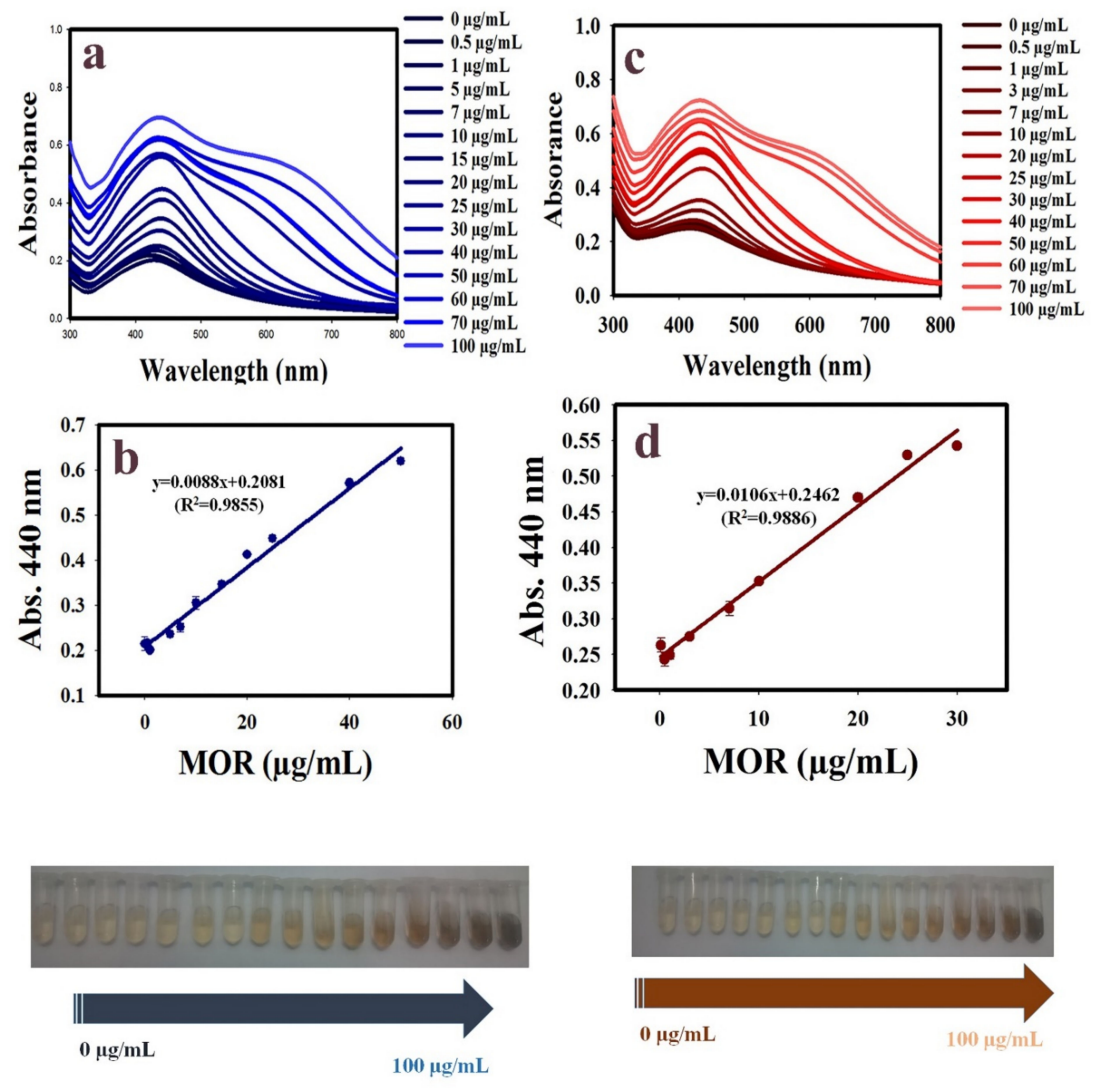
UV-visible spectra of (**a**,**b**) Au@Ag (P) solutions with different concentrations of MOR, calibration graphs for the quantification of MOR by using Au@Ag (P) as a colorimetric probe, and photographic images of Au@Ag (P) in the concentration range of 0−100 µg/mL; (**c**,**d**) Au@Ag (B) solutions with different concentrations of MOR, calibration graphs for the quantification of MOR by using Au@Ag (B) as a colorimetric probe, and photographic images of Au@Ag (B) in the concentration range of 0–100 µg/mL.

A typical calibration curve was determined under optimal conditions to detect MOR. The calibration curves exhibited a linear range of 0–50 and 0–30 µg/mL with y = 0.0088x + 0.2081 (R^2^ = 0.9855), and y = 0.0106x + 0.2462 (R^2^ = 0.9886), with a LOD of 0.100 µg/mL and 0.055 µg/mL for Au@Ag (P) and Au@Ag (B), respectively (Figure 8). A comparison between this method and the other methods is shown in Table 3.

### 3.5. Interference Effect

A proper Au@Ag NPs chemosensor must selectively bind to MOR in the presence of interfering species. The specificity and selectivity of the Au@Ag NPs were explored through a comparison of their color variations upon separate exposure to several drugs [methadone (MTD), acetaminophen (AP), tramadol (TRA), methylphenidate (MEPS), and codeine (COD)] and some potentially interfering substances in urine samples including various cations and anions (Ca^2+^, Zn^2+^, Na^+^, SO_4_^2−^, Cl^−^, CO_3_^2−^), and urea.

The choice of the mentioned drugs is because some of them (MEPS, TRA, and MTD) have been commonly consumed along with other over-the-counter drugs to reach similar analgesic effects.

To this end, these compounds were separately added to Au@Ag NPs colloidal solution (100 µg/mL). Results show that ions with a concentration of 100 µg/mL did not interfere.

**Figure 9:**
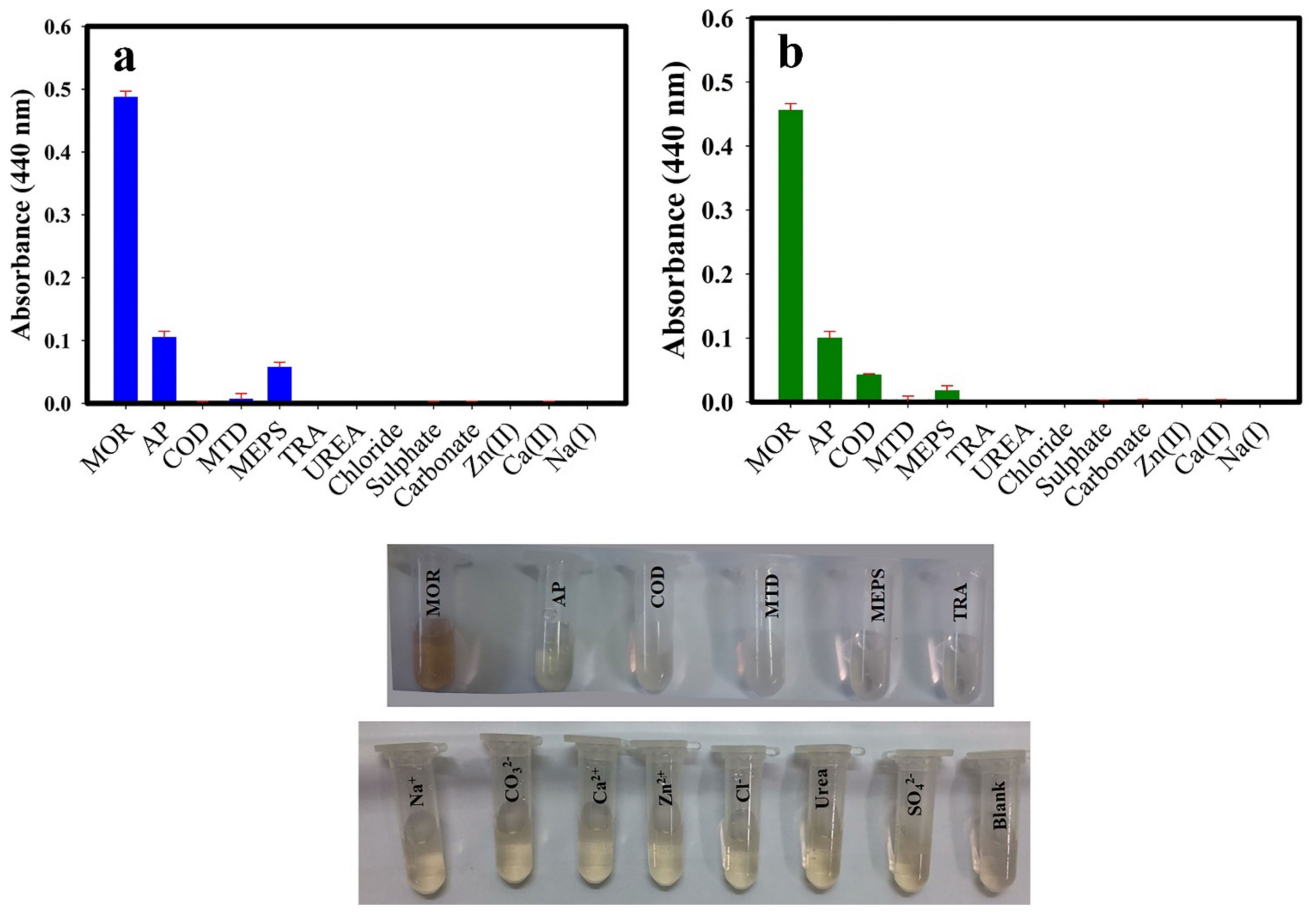
(**a**) UV-visible spectra of (**a**) Au@Ag (P) and (**b**) Au@Ag (B) NPs solutions with different drugs, urea, cations, and anions where the concentration of drugs and all other interferences was 100 µg/mL, and related photographic images.

Figure 9 shows a significant color variation upon MOR addition with maximal shifts from 420 nm to 440 nm. Figure S1 also represents the chemical structure of other drugs. The hydroxyl groups and the furan-like oxygen ring allow MOR to have hydrogen bonds and donor-acceptor electron interactions with the Au@Ag NPs surface. Among the other drugs, only AP has one OH group and exhibited a weak interaction with Au@Ag NPs. Additionally, codein (COD) and MOR have similar structures, with COD having a O-CH_3_ instead of OH group in MOR (Figure S1). This subtle difference can lead to selective interaction between MOR and Au@Ag NPs.

### 3.6. Real Sample

The applicability of the proposed method in a real sample was assessed in determining the MOR content in spiked urine samples. The standard addition procedure was applied in order to circumvent the matrix effects. The urine sample was pretreated according to the procedure described in the Experimental section, and certain amounts of the drugs, which were previously verified to lie in the known linear range, individually spiked. UV-vis spectra of the solutions were measured after the incubation of 5 min. The obtained results showed good recoveries (87–104 ℅) (Table 4), suggesting the applicability of the proposed method for the detection of MOR.

### 3.7. Sensing Mechanism

**Scheme 2:**
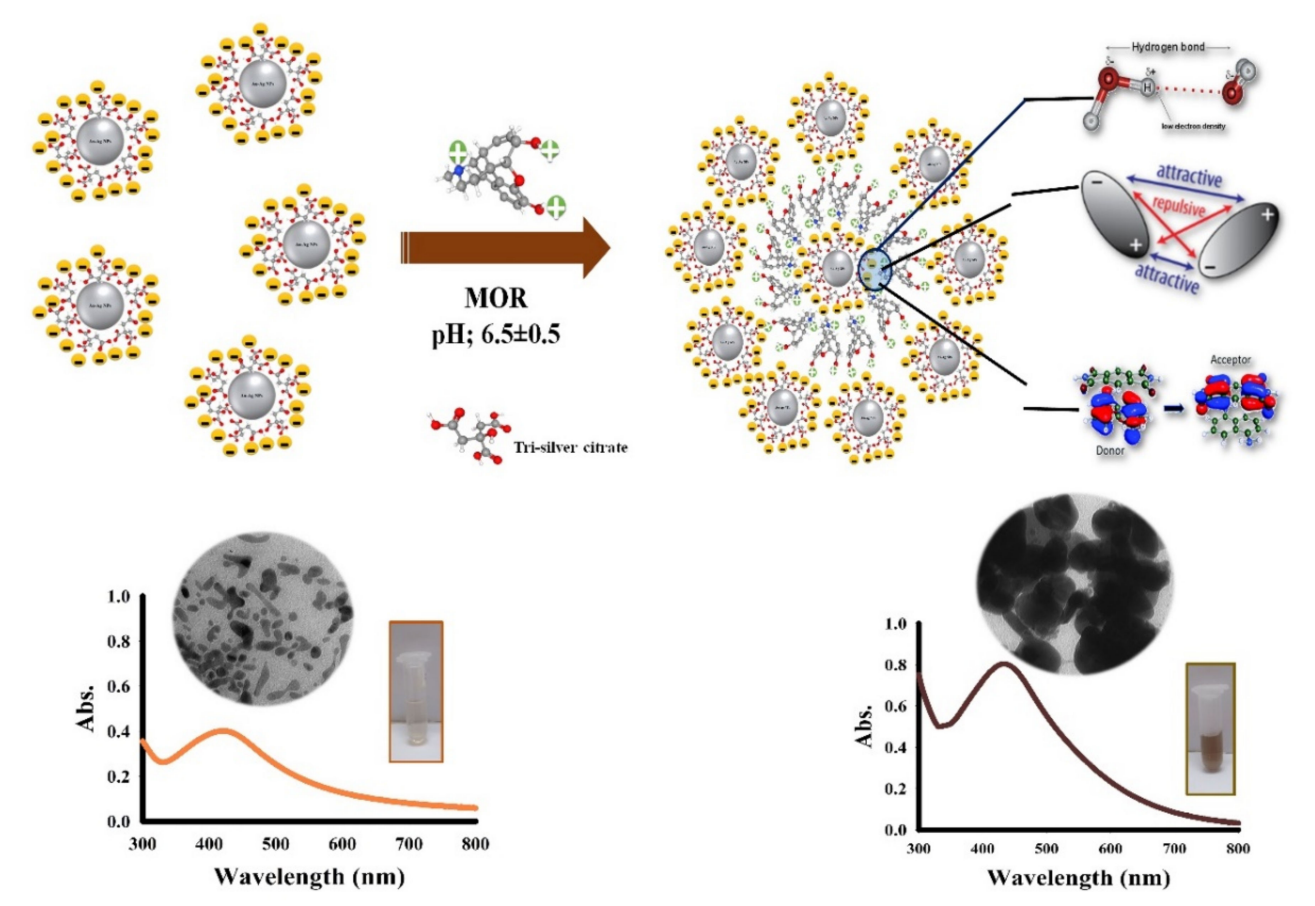
Illustration of the detection mechanism of MOR using Au@Ag NPs.

The sensing mechanism of MOR in presence of Au@Ag NPs can be described by the following mechanism. Based on the ζ potential of Au@Ag NPs, they have a negative surface charge due to the presence of silver citrate and their oxygen functional groups. The pKa of MOR ionization was reported 9.9 [46] and it has been reported that, at pH = 6.5 ± 0.5, MOR is in a non-ionized form [48]. Furthermore, hydroxyl groups and furan-like oxygen hybrid rings of MOR can form strong hydrogen bonds with trisilver citrate and glycerol on the surface of NPs. Therefore, MOR interaction with Au@Ag NPs involves hydrogen bonding, and donor-acceptor electron complex, explaining the rapid aggregation of NPs in the presence of MOR. Scheme 2 shows the proposed interaction mechanism between the Au@Ag NPs and MOR. 

## 4. Conclusions

A fast, facile, cost-effective, and selective chemosensor was developed for the quantitative detection of MOR in human-urine samples, based on the aggregation of Au@Ag NPs. The colorimetric nanoprobes were silver citrate-coated Au@Ag NPs synthesized by a sonochemical method. They showed a linear range of 0–50 µg/mL (R^2^ = 0.9855) and 0–30 µg/mL (R^2^ = 0.9886), with a LOD of 0.100 µg/mL and 0.055 µg/mL for Au@Ag NPs synthesized in presence of high-power and low-power ultrasonic irradiation, respectively. It is also worthwhile to note that no significant color changes were obtained in the presence of morphine using Au@Ag NPs synthesized without ultrasonic irradiation. From the obtained results in our research, we can see that the ultrasonic treatment dramatically improved the sensitivity for MOR determination. To the best of our knowledge, this is the first report on the colorimetric assay of MOR using sono-synthesized silver nanoparticles as a green method. Future work is now ongoing in our labs to explore a more selective narcotics detection.

## Figures and Tables

**Table 1:** Synthesis conditions for the Au@Ag NPs, and test results in the sensing of MOR.

Sample	Intensity (W/cm^2^)	Condition	Glycerol (M)	HAuCl_4_ (M)	AgNO_3_ (M)	Sodium Citrate (M)	λmax (nm)	λmax after Addition of Morphine (nm)
A_1_	26	Probe type	-	-	5.0 × 10^−3^	1.0 × 10^−2^	No significant peak	425
A_2_	26	Probe type	-	1.0 × 10^−5^	5.0 × 10^−3^	1.0 × 10^−2^	431	435
A_3_	26	Probe type	1	-	5.0 × 10^−3^	1.0 × 10^−2^	440	440
A_4_	26	Probe type	1	1.0 × 10^−5^	5.0 × 10^−3^	1.0 × 10^−2^	420	440
A_5_	21	Probe type	1	1.0 × 10^−5^	5.0 × 10^−3^	1.0 × 10^−2^	423	440
A_6_	30.5	Probe type	1	1.0 × 10^−5^	5.0 × 10^−3^	1.0 × 10^−2^	420	440
A_7_	<0.3	Bath type	1	1.0 × 10^−5^	5.0 × 10^−3^	1.0 × 10^−2^	426	440
A_8_	-	Stirrer method	1	1.0 × 10^−5^	5.0 × 10^−3^	1.0 × 10^−2^	No significant peak	435

**Table 2:** TEM and DLS analysis for Au@Ag NPs radius.

	TEM		DLS	
	NPs (nm)	NPs +MOR (nm)	NPs (nm)	NPs + MOR (nm)
Au@Ag (B)	5	39	36	380
Au@Ag (P)	10	60	39	114

**Table 3:** Comparison of detection method for MOR.

Method	Linear Range	LOD (mg/L)	Reference
Molecular imprinting-chemiluminescence	5 × 10^−9^–1 × 10^−6^ (g/mL)	2 × 10^−6^ (g/mL)	[54]
Spectrophotometry	0.07–7.98 (mg/L)	0.03 (mg/L)	[7]
GC- MS	0.05–5 (µg/g)	0.005 (µg/g)	[55]
GC-MS	5–500 (ng/mL)	1 (ng/mL)	[56]
GC–MS	0–750 (ng/mL)	0.75 (ng/mL)	[57]
HPLC-MS	50–1600 (ng/mL)	40 (ng/mL)	[58]
Chemical sensor-Colorimetric method Citrate-capped gold nanoparticles	1.33–33.29 (µg/mL)	0.15 (µg/mL)	[59]
Chemical sensor-Colorimetric method Cr(III) Metal-Organic Framework	0.1–350 (nM)	0.167 (nM)	[49]
Chemical sensor-Colorimetric method Melamine modified gold nanoparticles (MA–AuNPs)	0.07–3.0 (µM)	17 (nM)	[14]
Au@Ag (P)	0–50 (µM)	0.1 (µg/mL)	Present work
Au@Ag (B)	0–30 (µM)	0.055 (µg/mL)	Present work

**Table 4:** Analysis of MOR in spiked urine samples by the proposed method (incubation time: 5 min).

Sample	Added	Found	Recovery (%)	Std.Dev **
	(µg/mL)	(µg/mL)		
Au@Ag (P)	0	ND *	-	-
Au@Ag (P)	50	45.6	91.2	0.127
Au@Ag (P)	30	26.6	88.6	0.212
Au@Ag (P)	1	0.87	87	0.167
Au@Ag (B)	0	ND	-	-
Au@Ag (B)	30	31.4	104	0.141
Au@Ag (B)	10	9.7	97	0.112
Au@Ag (B)	1	1.01	101	0.099

* ND: not detected. ** Std.Dev (standard deviation value for duplicate measurements).

## Supplementary Materials

The following supporting information can be downloaded at: https://www.mdpi.com/article/10.3390/s22052072/s1, Figure S1: Chemical structure of drugs. Table S1: Physicochemical properties of morphine.

## References

[1] Gholivand M.-B., Jalalvand A.R., Goicoechea H.C., Gargallo R., Skov T., Paimard G. (2015). Combination of electrochemistry with chemometrics to introduce an efficient analytical method for simultaneous quantification of five opium alkaloids in complex matrices. Talanta.

[2] Paul B.D., Mell L.D., Mitchell J.M., Irving J., Novak A.J. (1985). Simultaneous Identification and Quantitation of Codeine and Morphine in Urine by Capillary Gas Chromatography and Mass Spectroscopy. J. Anal. Toxicol..

[3] Wallace J.E., Harris S.C., Peek M.W. (1980). Determination of morphine by liquid chromatography with electrochemical detection. Anal. Chem..

[4] Zhang C., Han Y., Lin L., Deng N., Chen B., Liu Y. (2017). Development of Quantum Dots-Labeled Antibody Fluorescence Immunoassays for the Detection of Morphine. J. Agric. Food Chem..

[5] Merchaoui S., Ben Said A., Louati K., Hajri A., Safta F., Kallel M. (2019). Optimization of morphine extraction method for the assay of its urinary 3-glucuronideconjuguate by gas chromatography-mass spectrometry. Ann. Pharm. Franc..

[6] Maccaferri G., Terzi F., Xia Z., Vulcano F., Liscio A., Palermo V., Zanardi C. (2019). Highly sensitive amperometric sensor for morphine detection based on electrochemically exfoliated graphene oxide. Application in screening tests of urine samples. Sens. Actuators B Chem..

[7] Sheibani A., Shishehbore M.R., Mirparizi E. (2010). Kinetic spectrophotometric method for the determination of morphine in biological samples. Spectrochim. Acta Part A Mol. Biomol. Spectrosc..

[8] Espinosa Bosch M., Ruiz Sanchez A., Anchez Rojas F.S., Bosch Ojeda C. (2007). Morphine and its metabolites: Analytical methodologies for its determination. J. Pharm. Biomed. Anal..

[9] Sverrisdóttir E., Lund T.M., Olesen A.E., Drewes A.M., Christrup L.L., Kreilgaard M. (2015). A review of morphine and morphine-6-glucuronide’s pharmacokinetic–pharmacodynamic relationships in experimental and clinical pain. Eur. J. Pharm. Sci..

[10] Ahmed S.R., Chand R., Kumar S., Mittal N., Srinivasan S., Rajabzadeh A.R. (2020). Recent Biosensing Advances in the Rapid Detection of Illicit Drugs. Trends Anal. Chem..

[11] Masteri-Farahani M., Mosleh N. (2019). Functionalization of graphene quantum dots with antimorphine: Design of selective nanosensor for detection of morphine. Mater. Lett..

[12] Masteri-Farahani M., Khademabbasi K., Mollatayefeh N. (2018). A selective morphine nanosensor derived from functionalized CdS quantum dots. Mater. Lett..

[13] Vickers N.J. (2017). Animal Communication: When I’m Calling You, Will You Answer Too?. Curr. Biol..

[14] Mohseni N., Bahram M. (2016). Mean centering of ratio spectra for colorimetric determination of morphine and codeine in pharmaceuticals and biological samples using melamine modified gold nanoparticles. Anal. Methods.

[15] Darroudi M., Khorsand Zak A., Muhamad M.R., Huang N.M., Hakimi M. (2012). Green synthesis of colloidal silver nanoparticles by sonochemical method. Mater. Lett..

[16] Ary K., Róna K. (2001). LC determination of morphine and morphine glucuronides in human plasma by coulometric and UV detection. J. Pharm. Biomed. Anal..

[17] Stojadinovic U., Matenco L., Andriessen P., Toljić M., Rundić L., Ducea M.N. (2017). Structure and provenance of Late Cretaceous–Miocene sediments located near the NE Dinarides margin: Inferences from kinematics of orogenic building and subsequent extensional collapse. Tectonophysics.

[18] Yu L., Song Z., Peng J., Yang M., Zhi H., He H. (2020). Progress of gold nanomaterials for colorimetric sensing based on different strategies. TrAC Trends Anal. Chem..

[19] Rohani Bastami T., Ghaedi A., Mitchell S.G., Javadian-Saraf A., Karimi M. (2020). Sonochemical synthesis of polyoxometalate-stabilized gold nanoparticles for point-of-care determination of acetaminophen levels: Preclinical study in an animal model. RSC Adv..

[20] Rohani Bastami T., Dabirifar Z. (2020). AuNPs@ PMo 12 nanozyme: Highly oxidase mimetic activity for sensitive and specific colorimetric detection of acetaminophen. RSC Adv..

[21] Jouyban A., Rahimpour E. (2020). Optical sensors based on silver nanoparticles for determination of pharmaceuticals: An overview of advances in the last decade. Talanta.

[22] Ibrahim N., Jamaluddin N.D., Tan L.L., Mohd Yusof N.Y. (2021). A Review on the Development of Gold and Silver Nanoparticles-Based Biosensor as a Detection Strategy of Emerging and Pathogenic RNA Virus. Sensors.

[23] Zhang F., Zhu J., Li J.-J., Zhao J.-W. (2015). A promising direct visualization of an Au@Ag nanorod-based colorimetric sensor for trace detection of alpha-fetoprotein. J. Mater. Chem. C.

[24] Huang H., Li Q., Wang J., Li Z., Yu X.-F., Chu P.K. (2014). Sensitive and Robust Colorimetric Sensing of Sulfide Anion by Plasmonic Nanosensors Based on Quick Crystal Growth. Plasmonics.

[25] Park G., Lee C., Seo D., Song H. (2012). Full-Color Tuning of Surface Plasmon Resonance by Compositional Variation of Au@Ag Core–Shell Nanocubes with Sulfides. Langmuir.

[26] Gao Z., Deng K., Wang X.-D., Miró M., Tang D. (2014). High-Resolution Colorimetric Assay for Rapid Visual Readout of Phosphatase Activity Based on Gold/Silver Core/Shell Nanorod. ACS Appl. Mater. Interfaces.

[27] Al-Masoodi A.H.H., Nazarudin N.F.F.B., Nakajima H., Tunmee S., Goh B.T., Majid W.H.B.A. (2020). Controlled growth of silver nanoparticles on indium tin oxide substrates by plasma-assisted hot-filament evaporation: Physical properties, composition, and electronic structure. Thin Solid Film..

[28] Alula M.T., Lemmens P., Bo L., Wulferding D., Yang J., Spende H. (2019). Preparation of silver nanoparticles coated ZnO/Fe_3_O_4_ composites using chemical reduction method for sensitive detection of uric acid via surface-enhanced Raman spectroscopy. Anal. Chim. Acta.

[29] Rivera-Rangel R.D., González-Muñoz M.P., Avila-Rodriguez M., Razo-Lazcano T.A., Solans C. (2018). Green synthesis of silver nanoparticles in oil-in-water microemulsion and nano-emulsion using geranium leaf aqueous extract as a reducing agent. Colloids Surf. A Physicochem. Eng. Asp..

[30] Banu R., Ramakrishna D., Reddy G.B., Veerabhadram G., Mangatayaru K.G. (2021). Facile one-pot microwave-assisted green synthesis of silver nanoparticles using Bael gum: Potential application as catalyst in the reduction of organic dyes. Mater. Today Proc..

[31] Joy Prabu H., Johnson I. (2015). Plant-mediated biosynthesis and characterization of silver nanoparticles by leaf extracts of Tragia involucrata, Cymbopogon citronella, Solanum verbascifolium and Tylophora ovata. Karbala Int. J. Mod. Sci..

[32] Mohammadi Z., Entezari M.H. (2018). Sono-synthesis approach in uniform loading of ultrafine Ag nanoparticles on reduced graphene oxide nanosheets: An efficient catalyst for the reduction of 4-Nitrophenol. Ultrason. Sonochem..

[33] Rohani Bastami T., Entezari M.H. (2012). A novel approach for the synthesis of superparamagnetic Mn_3_O_4_ nanocrystals by ultrasonic bath. Ultrason. Sonochem..

[34] Okitsu K., Ashokkumar M., Grieser F. (2005). Sonochemical Synthesis of Gold Nanoparticles:  Effects of Ultrasound Frequency. J. Phys. Chem. B.

[35] Anandan S., Grieser F., Ashokkumar M. (2008). Sonochemical Synthesis of Au−Ag Core−Shell Bimetallic Nanoparticles. J. Phys. Chem. C.

[36] Rohani Bastami T., Entezari M.H. (2013). High stable suspension of magnetite nanoparticles in ethanol by using sono-synthesized nanomagnetite in polyol medium. Mater. Res. Bull..

[37] Golsheikh A.M., Lim H.N., Zakaria R., Huang N.M. (2015). Sonochemical synthesis of reduced graphene oxide uniformly decorated with hierarchical ZnS nanospheres and its enhanced photocatalytic activities. RSC Adv..

[38] Vinodgopal K., Neppolian B., Lightcap I.V., Grieser F., Ashokkumar M., Kamat P.V. (2010). Sonolytic Design of Graphene−Au Nanocomposites. Simultaneous and Sequential Reduction of Graphene Oxide and Au(III). J. Phys. Chem. Lett..

[39] Wang X.-K., Shao L., Guo W.-L., Wang J.-G., Zhu Y.-P., Wang C. (2009). Synthesis of dendritic silver nanostructures by means of ultrasonic irradiation. Ultrason. Sonochem..

[40] Yao T., Sun Z., Li Y., Pan Z., Wei H., Xie Y., Nomura M., Niwa Y., Yan W., Wu Z. (2010). Insights into Initial Kinetic Nucleation of Gold Nanocrystals. J. Am. Chem. Soc..

[41] De Yoreo J.J., Vekilov P.G. (2003). Principles of Crystal Nucleation and Growth. Rev. Mineral. Geochem..

[42] Rohani Bastami T., Entezari M.H. (2012). Synthesis of manganese oxide nanocrystal by ultrasonic bath: Effect of external magnetic field. Ultrason. Sonochem..

[43] Li Z., Friedrich A., Taubert A. (2008). Gold microcrystal synthesis via reduction of HAuCl4 by cellulose in the ionic liquid 1-butyl-3-methyl imidazolium chloride. J. Mater. Chem..

[44] Xu J., Zhao T., Liang Z., Zhu L. (2008). Facile Preparation of AuPt Alloy Nanoparticles from Organometallic Complex Precursor. Chem. Mater..

[45] Bastús N.G., Merkoçi F., Piella J., Puntes V. (2014). Synthesis of Highly Monodisperse Citrate-Stabilized Silver Nanoparticles of up to 200 nm: Kinetic Control and Catalytic Properties. Chem. Mater..

[46] Wang M., Tan G., Ren H., Xia A., Liu Y. (2019). Direct double Z-scheme O-g-C3N4/Zn_2_SnO_4_N/ZnO ternary heterojunction photocatalyst with enhanced visible photocatalytic activity. Appl. Surf. Sci..

[47] Han S.W., Kim Y., Kim K. (1998). Dodecanethiol-Derivatized Au/Ag Bimetallic Nanoparticles: TEM, UV/VIS, XPS, and FTIR Analysis. J. Colloid Interface Sci..

[48] Wang X.-j., Li X., Yang S. (2009). Influence of pH and SDBS on the Stability and Thermal Conductivity of Nanofluids. Energy Fuels.

[49] Alhaddad M., Sheta M.S. (2020). Dual Naked-Eye and Optical Chemosensor for Morphine Detection in Biological Real Samples Based on Cr(III) Metal−Organic Framework Nanoparticles. ACS Omega.

[50] Chapman D.V., Way E.L. (1980). Metal Ion Interactions with Opiates. Annu. Rev. Pharmacol. Toxicol..

[51] Wang F., Lu Y., Chen Y., Sun J., Liu Y. (2018). Novel colorimetric nanosensor based on the aggregation of AuNP triggered by carbon quantum dots for detection of Ag^+^ ions. ACS Sustain. Chem. Eng..

[52] Roy S.D., Flynn G.L. (1989). Solubility Behavior of Narcotic Analgesics in Aqueous Media: Solubilities and Dissociation Constants of Morphine, Fentanyl, and Sufentanil. Pharm. Res..

[53] Traiwatcharanon P., Timsorn K., Wongchoosuk C. (2017). Flexible room-temperature resistive humidity sensor based on silver nanoparticles. Mater. Res. Express.

[54] He Y., Lu J., Liu M., Du J., Nie F. (2005). Determination of morphine by molecular imprinting-chemiluminescence method. J. Anal. Toxicol..

[55] Kudo K., Ishida T., Nishida N., Yoshioka N., Inoue H., Tsuji A., Ikeda N. (2006). Simple and sensitive determination of free and total morphine in human liver and kidney using gas chromatography–mass spectrometry. J. Chromatogr. B.

[56] Javidnia K., Miri R., Miri D. (2006). A Rapid and Sensitive Method for Determination of Morphine in Saliva of Opium Addicts. Iran. J. Med. Sci..

[57] Fryirs B., Dawson M., Mather L.E. (1997). Highly sensitive gas chromatographic–mass spectrometric method for morphine determination in plasma that is suitable for pharmacokinetic studies. J. Chromatogr. B.

[58] Zhang Y.R., Wang R., Zhang C.G. (2002). Column-switching high performance liquid chromatographic method for the determination of morphine and 6-monoacetylmorphine in urine. Shanghai Inst. Forensic Sci..

[59] Bahram M., Madrakian T., Alizadeh S. (2017). Simultaneous colorimetric determination of morphine and ibuprofen based on the aggregation of gold nanoparticles using partial least square. J. Pharm. Anal..

