# Supplementary material for: Naked-Eye Detection of Morphine by Au@Ag Nanoparticles-Based Colorimetric Chemosensors
Source: Sensors (Basel). 2022 Mar 7;22(5):2072. doi: 10.3390/s22052072 (PMC8914838; doi:10.3390/s22052072)
Supplement: Supplementary file 1 [file sensors-22-02072-s001.zip › sensors-1560961-supplementary.pdf]

Article

Naked-Eye Detection of Morphine by Au@Ag Nanoparticles-Based Colorimetric Chemosensors

Tahereh Rohani Bastami [1,*], Mansour Bayat [1] and Roberto Paolesse [2,*]

[1] Department of Chemical Engineering and Energy, Quchan University of Technology, Quchan 94771-67335, Iran; mansour.chem@gmail.com
[2] Department of Chemical Science and Technologies, University of Rome Tor Vergata, Via Della Ricerca Scientifica 1, 00133 Rome, Italy
* Correspondence: t.rohani@qiet.ac.ir (T.R.B.); roberto.paolesse@uniroma2.it (R.P.)

Supplementary Information

Table S1: Physicochemical properties of morphine [1].

Drug	Chemical name and formula	Structure	So H_2O (25°C $mg.mL^{-1}$)	pKa
Morphine sulfate salt pentahydrate,	7,8-Didehydro-4,5a-epoxy-17-methylmorphinan-3,6-a-diol sulfate (2:1 salt) $C_{34}H_{40}N_2O_{10}S \cdot 5H_2O$	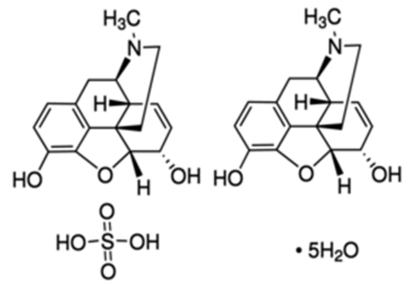	64	8.5

References

1. Sheibani, A.; Shishehbore, M.R.; Mirparizi, E. Kinetic spectrophotometric method for the determination of morphine in biological samples. *Spectrochim. Acta Part A Mol. Biomol. Spectrosc.* **2010**, *77*, 535–538.

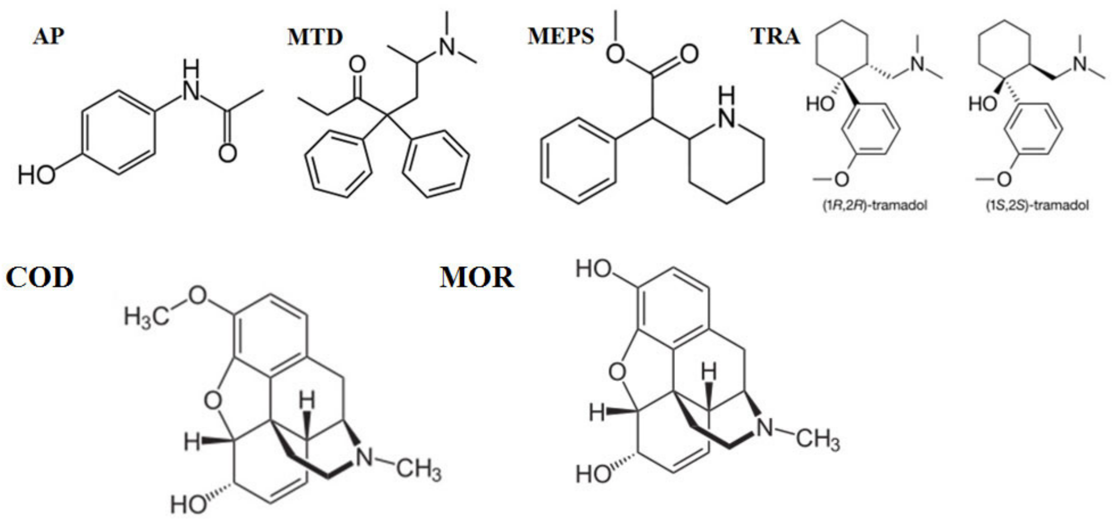

Figure S1. Chemical structure of drugs
